# The Role of Nailfold Capillaroscopy in Evaluating Patients with Interstitial Lung Disease Related to Connective Tissue Disease

**DOI:** 10.31138/mjr.20230804.tr

**Published:** 2023-08-04

**Authors:** Aliki I. Venetsanopoulou, Andreas V. Goules, Panayiotis G. Vlachoyiannopoulos, Alexandros A. Drosos, Athanasios G. Tzioufas, Paraskevi V. Voulgari

**Affiliations:** 1 Department of Rheumatology, School of Health Sciences, Faculty of Medicine, University of Ioannina, Greece,; 2 Department of Pathophysiology, School of Medicine, National and Kapodistrian University of Athens, Greece,; 3 Department of Pathophysiology, School of Medicine, National and Kapodistrian University of Athens, Greece; Institute for Autoimmune Systemic and Neurologic Diseases, Athens, Greece

**Keywords:** capillaroscopy, interstitial lung disease, autoimmune diseases, scleroderma

## Abstract

Capillaroscopy is a non-invasive and safe imaging method that allows the evaluation of the microcirculation of the small vessels of the skin. The method’s main advantage is the early detection of microvascular changes that may occur in certain connective tissue diseases (CTDs). Today, the presence of specific autoantibodies and capillaroscopic findings are generally accepted and emerge as a powerful diagnostic tool for detecting underlying CTDs in patients with Raynaud’s phenomenon. The role of capillaroscopy has also been investigated in patients with CTD and interstitial lung disease (ILD). In these patients, lung involvement is considered one of the most severe complications, potentially leading to significant morbidity and mortality. So far, studies have shown an association of the scleroderma pattern in capillaroscopy with lung involvement in Scleroderma patients. Although there are studies on the association of capillary findings in patients with other CTDs, further efforts are needed to evaluate this technique and produce high-performance algorithms in the early detection of involvement and the progression of (CTD) related ILD (CTD-ILD). The present study aims to perform capillaroscopy in CTDILD patients with different imaging patterns and to correlate the method’s findings with those found in high-resolution computed tomography, pulmonary tests, and the immunological profile of patients. Furthermore, the impact of ILD treatment on the capillaroscopic findings will be evaluated.

## INTRODUCTION

Interstitial lung disease (ILD), a severe complication in patients with an underlying Connective tissue disease (CTD), disrupts lung function and patients’ overall physical capacity, leading to increased morbidity and mortality.^1,2^ CTDs related to ILD (CTD-ILD) include rheumatoid arthritis (RA), systemic lupus erythematosus (SLE), systemic sclerosis (Scl), Sjogren’s syndrome (SS), idiopathic inflammatory myopathies (polymyositis, dermatomyositis), mixed connective tissue disease (MCTD), undifferentiated connective tissue disease and ANCA-associated vasculitis. Of these diseases, Scl, RA, and myositis have the highest incidence of ILD.^3–7^ Non-specific interstitial pneumonia (NSIP) is the most commonly found radiological and histopathological pattern observed in CTD-ILDs, except for RA, where usual interstitial pneumonia (UIP) is more common than NSIP.^8^ Other patterns include lymphoid interstitial pneumonia (LIP),^9^ desquamative interstitial pneumonia (DIP), and organizing pneumonia (OP).^10^

So far, in clinical practice, there are two valuable diagnostic tools for detecting and evaluating progressing pulmonary disease: high-resolution (HR) computed tomography (CT) and lung function tests.^11^ Still, the need for early detection of patients who will develop lung involvement and those who already have and will likely present a faster deterioration remains.

Capillaroscopy, a non-invasive diagnostic technique designed to evaluate small microcirculation vessels, has been used to assess lung involvement in patients with Scl and other CTDs. Until recently, the main use of this technique was for distinguishing patients with primary Raynaud’s phenomenon from those with secondary due to Scl.^12^ Cutolo et al. have described the ‘early’, ‘active’, and ‘late’ Scl motifs using a high magnification technique.^13^ Since then, several researchers have used this subcategorization to correlate changes in capillaroscopy findings with organ involvement and disease severity in Scl.^14–16^ The observed alterations in the nailfold capillaroscopy have also been used to evaluate microvascular endothelial dysfunction in patients with other CTDs^17,18^ and patients with Pulmonary arterial hypertension associated with CTDs. ^19,20^

Regarding the evaluation of capillary findings specifically for CTD-ILD, the data from the literature are early but very encouraging. In 2013 a pilot study by Smith et al. showed that capillaroscopy patterns correlated with future severe, peripheral, vascular, and pulmonary involvement at 18-24 months of patients’ follow-up.^21^ The capillaroscopy pattern has been associated with ILD and cardiopulmonary involvement, regardless of the presence of antibodies to extractable nuclear antigens (anti-ENA) in these patients.^22^

In addition to patients with Scl, studies show that capillaroscopic findings should also be evaluated in patients with MCTD. In these patients, the presence of giant capillaries may be a future marker, particularly in those with short disease duration (<1 year).^23^ Furthermore, some studies have compared capillaroscopic findings between patients with different patterns of ILD. According to those, more severe capillary abnormalities were observed in patients with RA-ILD, idiopathic pulmonary fibrosis, and SS-ILD, while the mean capillary density in patients with the UIP pattern was decreased compared to NSIP patients.^24^

Based on the above, it is evident that using capillaros-copy as a non-invasive method, which could predict the involvement and progression of CTD-ILD, would be beneficial. Such an imaging modality could also be a strong indication for performing high-resolution computed tomography (HRCT) and probably for distinguishing patients who are most likely to benefit from the early initiation of specific immunosuppressive treatments.

## AIMS OF THE STUDY

This study aims to evaluate the role of capillaroscopy in a series of patients with CTD-ILD, including patients with RA, SS, Scl, and myositis. The findings of capillaroscopy will be correlated with the patients’ immunological profiles and the imaging and functional tests that will be performed. As there are few literature reports worldwide, the present study will provide essential conclusions about the different patterns of capillary findings depending on the underlying CTD and the type of lung involvement. Also, the study will explore the predictive value of capillaroscopy as an indicator of lung disease progression in patients with CTD-ILD and response to the received treatment. The results are likely to impact both the evaluation and treatment of these patients.

## METHODS

The study will be prospective and involve patients diagnosed with CTD-ILD who will attend two university medical centers in Greece. Demographic data, clinical characteristics, medication received, and immunological profile will be recorded in detail at the initial assessment. Imaging of the lung parenchyma will be performed with an HRCT, while lung function tests will be evaluated with spirometry and a test of the diffusing capacity of the lungs for carbon monoxide (DLCO).

A capillaroscopy will be performed based on a specific protocol: Before the examination, patients will remain in a room with a temperature of 20-22 ° C for 15-20 minutes. They will be instructed not to smoke and not to consume caffeine for at least 4 hours before the examination. Capillaroscopy will not be performed if a patient has recently (within the last 3 weeks) undergone any aesthetic intervention involving the nail area, as subsequent microtraumas can give false positive results.

The examination will concern the fingers of both hands (except the thumbs). One drop of special oil will be placed on the nail’s base to increase the skin’s transparency. Next, the entire nail area will be examined, and any abnormal findings will be recorded.

Based on capillaroscopy, the following parameters will be evaluated: The number of capillaries per 1 mm (capillary density), the size, shape, and architecture of the capillaries, pathological findings such as the presence of giant capillaries, and bleedings.

The study will use a particular video capillaroscopy with a magnification lens *200 to better visualize the capillaries and their morphological characteristics.

Patients will be examined 6-12 months after their initial visit with a new capillaroscopy, HRCT, and function lung tests to evaluate ILD progression and treatment response.

After data collection, statistical analysis and comparison of the findings will be made between the different CTDILD groups.

### Statistical Analysis

Microsoft Excel 2017 and Statistica v.12 will be used for the analysis. The data will be analysed using descriptive statistics: The continuous variables will be described by their mean and median values and their constant deviations and range. Categorical variables will be described as percentages of the total available records. Finally, a multivariate analysis will be performed to identify the independent risk factors for each variable.

### Study approval

The study has been approved by the Ethics Committee of the University Hospital of Ioannina.

## ABBREVIATIONS

Anti-ENA: Antibodies to Extractable Nuclear antigens
CTD: Connective tissue disease
CT: Computed tomography
DIP: Desquamative interstitial pneumonia
DLCO: Diffusing capacity of the lungs for carbon monoxide
HR: High-resolution
ILD: Interstitial lung disease
LIP: Lymphoid interstitial pneumonia
MCTD: Mixed connective tissue disease
NSIP: Non-specific interstitial pneumonia
RA: Rheumatoid arthritis
SLE: systemic lupus erythematosus
Scl: Systemic sclerosis
SS: Sjogren syndrome
OP: Organising pneumonia
UIP: Usual interstitial pneumonia
